# Profiling Inflammatory Extracellular Vesicles in Plasma and Cerebrospinal Fluid: An Optimized Diagnostic Model for Parkinson’s Disease

**DOI:** 10.3390/biomedicines9030230

**Published:** 2021-02-25

**Authors:** Elena Vacchi, Jacopo Burrello, Alessio Burrello, Sara Bolis, Silvia Monticone, Lucio Barile, Alain Kaelin-Lang, Giorgia Melli

**Affiliations:** 1 Laboratory for Biomedical Neurosciences, Neurocenter of Southern Switzerland, Ente Ospedaliero Cantonale, 6900 Lugano, Switzerland; elena.vacchi@eoc.ch (E.V.); alain.kaelin@eoc.ch (A.K.-L.); 2 Faculty of Biomedical Sciences, Universita della Svizzera Italiana, 6900 Lugano, Switzerland; lucio.barile@cardiocentro.org; 3 Laboratory for Cardiovascular Theranostics, Cardiocentro Ticino Institute, Ente Ospedaliero Cantonale, 6900 Lugano, Switzerland; jacopo.burrello@gmail.com (J.B.); sara.bolis@cardiocentro.org (S.B.); 4 Department of Electrical, Electronic and Information Engineering “Guglielmo Marconi”, University of Bologna, 40136 Bologna, Italy; alessio.burrello@unibo.it; 5 Division of Internal Medicine and Hypertension Unit, Department of Medical Sciences, University of Torino, 10126 Torino, Italy; smv.monticone@gmail.com; 6 Institute of Life Sciences, Scuola Superiore Sant’Anna, 56127 Pisa, Italy; 7 Department of Neurology, Neurocenter of Southern Switzerland, Ente Ospedaliero Cantonale, 6900 Lugano, Switzerland; 8 Department of Neurology, Inselspital, Berne University Hospital, 3010 Berne, Switzerland

**Keywords:** plasma, cerebrospinal fluid, extracellular vesicles, machine learning, biomarkers, Parkinson’s disease, multiple system atrophy, tauopathies, neuroinflammation

## Abstract

Extracellular vesicles (EVs) play a central role in intercellular communication, which is relevant for inflammatory and immune processes implicated in neurodegenerative disorders, such as Parkinson’s Disease (PD). We characterized and compared distinctive cerebrospinal fluid (CSF)-derived EVs in PD and atypical parkinsonisms (AP), aiming to integrate a diagnostic model based on immune profiling of plasma-derived EVs via artificial intelligence. Plasma- and CSF-derived EVs were isolated from patients with PD, multiple system atrophy (MSA), AP with tauopathies (AP-Tau), and healthy controls. Expression levels of 37 EV surface markers were measured by a flow cytometric bead-based platform and a diagnostic model based on expression of EV surface markers was built by supervised learning algorithms. The PD group showed higher amount of CSF-derived EVs than other groups. Among the 17 EV surface markers differentially expressed in plasma, eight were expressed also in CSF of a subgroup of PD, 10 in MSA, and 6 in AP-Tau. A two-level random forest model was built using EV markers co-expressed in plasma and CSF. The model discriminated PD from non-PD patients with high sensitivity (96.6%) and accuracy (92.6%). EV surface marker characterization bolsters the relevance of inflammation in PD and it underscores the role of EVs as pathways/biomarkers for protein aggregation-related neurodegenerative diseases.

## 1. Introduction

A major current medical need in the field of neurology is the identification of reliable biomarkers in vivo for the diagnosis of neurodegenerative diseases. Parkinson’s Disease (PD) is the second most common neurodegenerative disorder of the elderly, characterized by the progressive loss of dopaminergic neurons in the substantia nigra [1]. To date, an effective causal treatment is missing, and the diagnosis still relies exclusively on clinical evaluation of motor symptoms that appear late, when approximately 70% of dopaminergic neurons are already lost [2]. Thus, there is urgent need for accessible and reliable biomarkers that can stratify PD patients in clinical trials for novel therapies. Furthermore, PD and atypical parkinsonisms (AP) are often misdiagnosed. AP refers to a variety of protein aggregation-related diseases in which patients show signs and symptoms similar to PD but generally do not respond to dopaminergic drug treatment specific for PD and experience a more aggressive course of disease. According to the misfolded protein aggregates present in brain of patients, AP can be distinct in AP with synucleinopathies (AP-Syn), such as multisystem atrophy (MSA) and AP with tauopathies (AP-Tau), such as progressive supranuclear palsy (PSP) and corticobasal degeneration (CBD). The differential diagnosis between PD and AP is challenging [3], preventing an early and appropriate treatment.

Extracellular vesicles (EVs) are emerging as novel and sensitive biomarkers for neurodegenerative diseases [4,5]. EVs are a heterogeneous population of membrane particles involved in physiological cell-to-cell communication and transmission of biological signals. EVs are released into body fluids where they are easily accessible. EVs are secreted by many cell types [6,7,8]: they carry membrane receptors which allow the tracking of the cell of origin [9], while their protein and transcriptomic contents provide information on the cell physio-pathological state [10,11]. Central nervous system (CNS) neurons and glia cells release EVs [6] that are able to cross the blood–brain barrier and reach the peripheral blood [11].

In a previous work, we demonstrated that distinctive pools of plasma-derived EV surface markers related to inflammatory and immune cells stratified patients with PD and AP according to the clinical diagnosis. We characterized distinctive EV subpopulations in plasma by simultaneously immunophenotyping 37 different membrane proteins using a flow cytometry multiplex bead-based platform [12,13] and then a diagnostic model based on the distinctive EV surface proteins profile was generated via supervised machine learning algorithms. The resulting diagnostic model was able to correctly classify 88.9% of patients, with reliable diagnostic performance after internal and external validations [14]. Indeed, recent studies trying to dissect the cell/tissue origin of circulating EVs, showed that the majority of plasma-derived EVs come from hematopoietic cells, in particular platelets, B cells and T cells [15]. Thus, in order to increase the sensitivity and specificity of the model for neurodegenerative diseases, we analyzed matched samples of cerebrospinal fluid (CSF) and plasma in a subgroup of patients, assuming that EV surface markers, expressed both in plasma and CSF, may have higher relevance for the respective neurodegenerative disease. In fact, CSF permeates the cerebral cortex, spinal cord, cerebral ventricles, and medullary canal, receiving EVs mainly from the CNS. However, despite its higher specificity for CNS, CSF is less accessible and more difficult to obtain compared with plasma. Moreover, the EV content in CSF is lower and the protocol for extracting EVs is technically more challenging, requiring concentration passages which are not generally required for plasma [16].

Therefore, the aims of the present study were: (1) to characterize CSF-derived EVs in healthy subjects and patients with PD, MSA and AP-Tau; (2) to compare EVs profiling in matched CSF and plasma samples of PD and AP patients; (3) to improve our previous diagnostic model, based on plasma-derived EVs by integrating information provided by CSF-derived EVs immunophenotyping.

## 2. Materials and Methods

### 2.1. Subjects

A total of 16 patients, for which matched plasma and CSF samples were analyzed in the current study, made up the optimization cohort: 4 idiopathic PD, 4 probable MSA and 4 probable AP-Tau, among which 3 had probable progressive supranuclear palsy and 1 had possible corticobasal degeneration. The plasma discovery cohort included 84 subjects (Table S1) [9]. Subjects were recruited from the movement disorder outpatient clinic at Neurocenter of Southern Switzerland in Lugano. Inclusion criteria for MSA, PSP and CBD were based on published diagnostic criteria [17,18,19]. Each subject underwent blood sampling and clinical evaluation, and a subgroup also underwent CSF sampling. Disease gravity was assessed by Hoen and Yahr scale (H&Y) and Movement Disorder Society—Unified Parkinson’s Disease Rating Scale (MDS-UPDRS); cognitive profile by Mini-Mental State Evaluation (MMSE) and Montreal Cognitive Assessment (MoCA); mood disorder by Beck Depression Inventory II (BDI-II) scale; REM sleep Behavior Disorder (RBD) by RBD screening questionnaire; olfactory function by olfactory test (Burghart Messtechnik GmbH, Wedel, Germany). Levodopa equivalent daily dose (LEDD) was calculated for PD and AP [20]. Patients were excluded from the analysis in case of significant comorbidities: diabetes, renal failure, thyroid pathology, vitamin B12 deficiency, HIV infection, syphilis, coagulopathy, fever, acute or chronic inflammatory diseases, and tumors. Finally, 4 subjects who underwent CSF collection in emergency room for acute headache were included in the study as HC, after CSF analysis showed normal parameters and other pathologies were excluded. The 16 patients for which matched plasma and CSF samples were analyzed in the current study made up the optimization cohort, whereas 84 patients from our previous study [14] made up the plasma discovery cohort and were used to test basic and integrated diagnostic models.

### 2.2. Plasma and CSF Preparation

**Figure 1:**
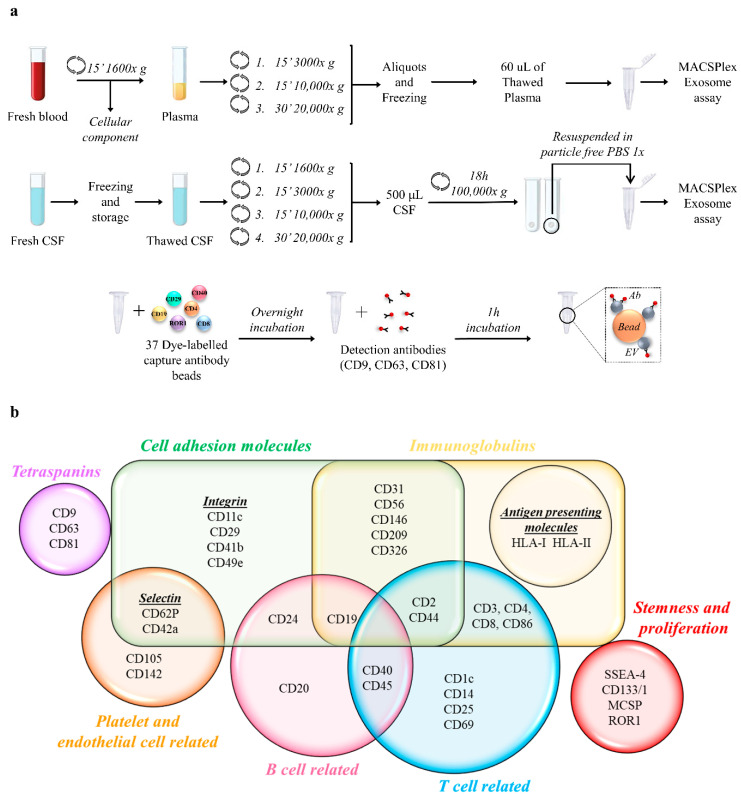
Plasma and CSF preparation for MACSPlex human Exosome assay. (**a**) Protocol for extracellular vesicle (EV) enrichment and characterization by MACSPlex Exosome Assay. Blood and cerebrospinal fluid (CSF) underwent serial centrifugation to eliminate cellular components and larger EVs. Samples were incubated overnight with phycoerythrin (PE)- and fluorescein isothiocyanate (FITC)-labeled capture beads, coated with antibodies against 37 different EV surface markers. APC-conjugated detection antibodies against CD9, CD63, and CD81 were added and incubated for 1 h. After washing steps, samples were analyzed by flow cytometry. (**b**) Schematic representation of the 37 EV surface markers analyzed by MACSPlex human Exosome assay.

In total, 10 mL of blood was collected into anticoagulant-EDTA tubes, after at least 4 h fasting. The following protocol was performed to obtain EV enriched plasma [21] (Figure 1a): samples were centrifuged for 15 min at 1600× *g* at 10 °C, to eliminate cellular components; then, three consecutive centrifuges were performed to further purify the plasma, eliminating apoptotic bodies and larger EVs (15 min at 3000× *g*, 15 min at 10,000× *g* and 30 min at 20,000× *g* at 4 °C). We previously demonstrated no significant change in flowcytometric analysis by MACSPlex assay of plasma samples with and without EV enrichment by ultracentrifugation [14]. Samples were aliquoted and stored at −80 °C.

Five mL of CSF was collected into 15 mL polypropylene tubes, and immediately frozen (Figure 1a). After thawing, 500 µL of CSF samples underwent serial centrifuges as plasma samples plus a further ultracentrifugation to maximize EV enrichment (18 h at 100,000× *g* at 4 °C). Pellets were resuspended in 30 µL of particle-free PBS. The storage period varied among samples according to the consecutive enrollment of subjects in the study, between 2015-07 and 2020-10.

### 2.3. Nanoparticle Tracking Analysis (NTA)

Nanoparticle concentrations and diameters were measured by NanoSight LM10 (Malvern Panalytical, Malvern, UK) equipped with a 405 nm laser and Nanoparticle Tracking Analysis NTA 2.3 software (Malvern Panalytical, Malvern, UK). Only for NTA analysis, to rule out a confounding effect of ultracentrifugation on EV amount and diameter, plasma samples (100 µL) were centrifuged akin to CSF samples for 18 h at 100,000× *g* at 4 °C. The obtained pellet was resuspended in 100 µL of particle-free PBS. A total of 1 µL of ultraconcentrated plasma or CSF was diluted in particle-free PBS—1:500 and 1:250, respectively. NTA analyses were performed as previously described [14].

### 2.4. MACSPlex Exosome Assay and Flow Cytometry Analysis

The screening approach (MACSPlex human Exosome Kit, Miltenyi, Bergisch Gladbach, Germany) was previously described [12,13] and is summarized in Figure 1a. The complete list of the 37 EV surface markers that were analyzed is represented in Figure 1b.

In total, 60 µL of plasma and 30 µL of ultracentrifuged CSF were added to the MACSPlex Buffer solution (final volume 120 µL) and analyzed with MACSQuant Analyzer-10 flow cytometer (Miltenyi, Bergisch Gladbach, Germany). As a blank control we used MACSPlex Buffer incubated with beads and detection antibodies. Median fluorescence intensity (MFI) for each EV surface marker was normalized by the mean MFI for specific EV markers (CD9, CD63, and CD81). All analyses were based on normalized MFI (nMFI) values. Samples were analyzed blind to the clinical diagnosis.

Tests for the reliability/specificity of MACSPlex human Exosome Kit for EVs and for the technical consistency and reproducibility of the assay were performed in our previous work [14].

### 2.5. Statistical Analysis

Statistics was performed using IBM SPSS Statistics 22.0 (IBM SPSS, Armonk, New York, NY, USA), PYTHON 2.7 (Python Software Foundation, DE, USA), and GraphPad PRISM 7.0a (GraphPad Software, San Diego, CA, USA). A Kolmogorov–Smirnov test was applied to evaluated variable distribution. Non-normally distributed variables (disease duration, H&Y, MDS-UPDRS, BDI-II, MMSE, MoCA, Olfactory test, RBD, LEDD, NTA, MACSPlex analysis) were expressed as medians (interquartile range) and analyzed by Kruskal–Wallis’ test. Normally distributed variables (age) were expressed as mean ± standard deviation (SD) and analyzed by 1-way ANOVA test with Bonferroni’s correction for multiple comparisons. Categorical variables (sex) were expressed as absolute number and percentage (%) and analyzed by χ² (when applicable) or Fisher’s tests. Matched measurements (plasma vs. CSF), variables were analyzed by Wilcoxon pairs signed rank test.

### 2.6. Diagnostic Modelling

Supervised learning algorithms were used to combine levels of expression of single EV surface markers in a specific EV surface marker signature and discriminate patients according to clinical diagnosis. Linear discriminant analysis was used as a feature reduction strategy to build the 3D canonical plots (Figure 3d,e); canonical components were calculated from the weighted linear combination of expression levels for the 37 EV markers in order to maximize the separation between the 4 groups (HC, PD, MSA, AP-Tau); each point represents a patient and spheres include patients with a linear combination coefficient that falls within the mean of canonical components 1, 2, and 3 ± SD. The diagnostic models were built exploiting a random forest (RF) classification algorithm, as previously reported [14,22,23]. Briefly, each forest is composed of 20 classification trees with a maximum number of 8 splits for each tree; the diagnosis derives from the outcome of each tree of the RF (i.e., if at least 11 of 20 trees of the RF predict PD, the patient will be classified as PD).

The basic RF models were built on the expression of 17 EV surface markers (CD29, CD45, CD19, CD42a, HLA-I, CD31, CD1x, CD11c, CD62P, CD40, CD41b, CD209, CD4, CD2, CD146, CD25, MCSP), which were demonstrated to be differentially expressed in plasma samples in HC, PD, MSA, and AP-Tau in our previous study [14]. Internal and external validation of the basic RF models is provided by Vacchi et al. [14].

The integrated version of RF models (see Figure 6) was built selecting only markers expressed in CSF- and plasma-derived EVs from patients with PD (8 EV markers: CD4, CD19, CD2, CD1c, HLA-I, CD41b, CD29, and CD45), MSA (10 EV markers: CD4, CD19, CD2, CD1c, HLA-I, MCSP, CD146, CD41b, CD29, and CD45), or AP-Tau (6 EV markers: CD4, CD2, CD1c, HLA-I, CD41b, and CD45). Integrated models were directly validated on the original discovery cohort. A representative classification tree was shown for each model, and a confusion matrix reported accuracy, sensitivity, specificity, positive and negative predictive values.

## 3. Results

### 3.1. Demographic and Clinical Characteristics

**Figure 2:**
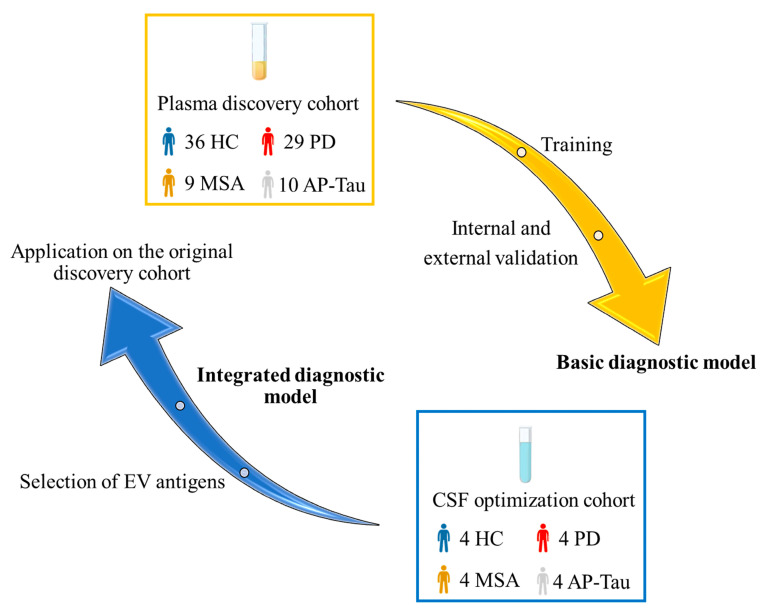
Study flowchart. The plasma discovery cohort was used to train and validate the basic diagnostic model based on plasma-derived extracellular vesicle (EV) profiling. The CSF-derived EV profiling in an optimization cohort was used to generate an integrated model that was subsequently tested in the discovery cohort.

The optimization cohort included 16 patients: 4 PD, 4 MSA, 4 AP-Tau (3 PSP, 1 CBD), and 4 healthy controls (HC). These subjects underwent plasma and CSF collection for paired flow cytometry analysis. Clinical characteristics are summarized in Table 1. No differences were observed between groups (*p* > 0.05 for all comparisons). The plasma discovery cohort included 84 patients from our previous study [14] and was used to test both the basic and integrated diagnostic models (Table S1). In Figure 2, a flowchart of the study is represented.

### 3.2. PD Group Shows an Increased Number of CSF-Derived EVs

**Figure 3:**
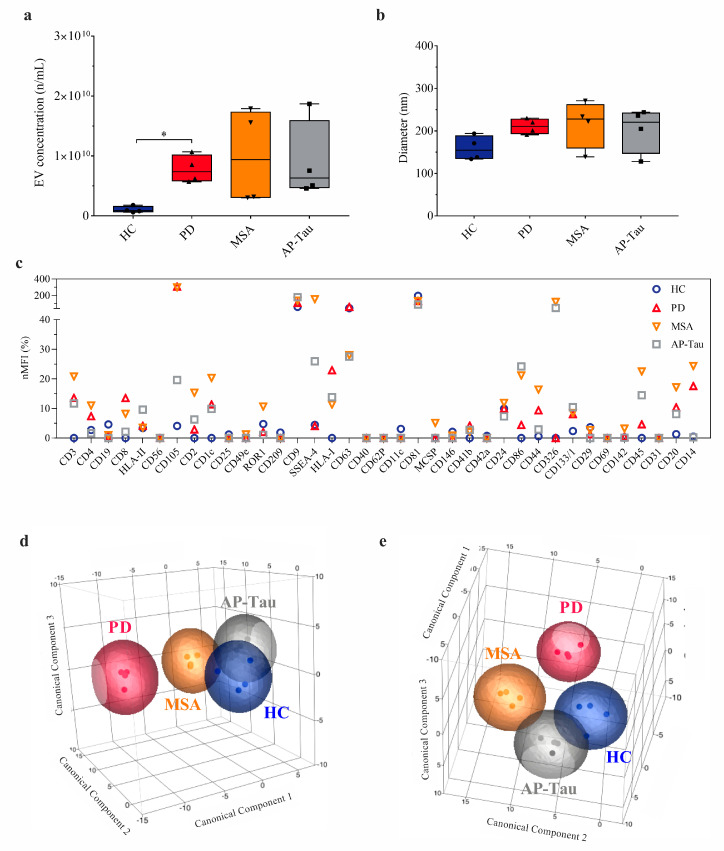
CSF-derived EVs characterization. Characterization of cerebrospinal fluid (CSF)-derived extracellular vesicles (EVs) by nanoparticle tracking analysis (NTA) and MACSPlex human exosome assay flow cytometry. Healthy controls (HC) were compared with patients with Parkinson’s disease (PD) multisystem atrophy (MSA), or atypical parkinsonism with tauopathies (AP-Tau). (**a**) EV concentration (n/mL CSF) at NTA. (**b**) EV diameter (nm) at NTA. Boxplots show median and interquartile range; bars show minimum and maximum values (* *p* < 0.05). (**c**) Normalized median fluorescence intensity (nMFI; %) for 37 EV surface markers. Data and statistics are reported in Table S1. (**d**,**e**) Different perspectives of 3D-canonical plot reporting patient discrimination according to EV surface marker expression (each patient is indicated by a point and diagnoses are represented by colors: HC, blue; PD, red; MSA, orange; AP-Tau, grey). Canonical axes of the plot (canonical components 1, 2, and 3) are defined by linear discrimination analysis from weighted linear combinations of the 37 EV markers analyzed by flow cytometry. Spheres include patients with linear combination coefficients that fall within the mean ± SD (canonicals 1, 2, and 3 ± SD).

Nanoparticle tracking analysis (NTA) revealed higher concentration of CSF-derived nanoparticles/mL in PD compared to HC (*p* = 0.048); this difference was mainly due to larger vesicles (151–500 nm; *p* = 0.045; Table S2). A trend towards a higher amount of EVs was observed also in MSA and AP-Tau. Although EV diameter was 1.4/1.5-fold higher in PD and AP patients compared to HC, the difference was not significant (Figure 3b).

### 3.3. CSF-Derived EV Immunophenotyping Stratifies Patients According to the Clinical Diagnosis

The immunophenotyping of 37 surface markers on CSF-derived EVs by MACSPlex assay did not identify statistically significant differences between the four groups when each EV marker was considered individually (Figure 3c and Table S2). However, several markers, related to T cells (CD2, CD3, CD8, CD14, CD86), B cells (CD20), endothelia cells (CD105) and the major histocompatibility complex class I human leukocyte antigen-ABC (HLA-ABC), displayed higher expressions in PD, MSA, AP-Tau then HC. Nevertheless, the linear weighted combination of all EV markers in a single specific signature by supervised learning (linear discriminant analysis, see methods) allowed the discrimination of patients according to their diagnosis, as shown in the canonical plots (Figure 3d,e).

### 3.4. Paired Analysis of CSF vs. Plasma in Patients Identifies Different Amount of EVs and Different Surface Marker Expressions

**Figure 4:**
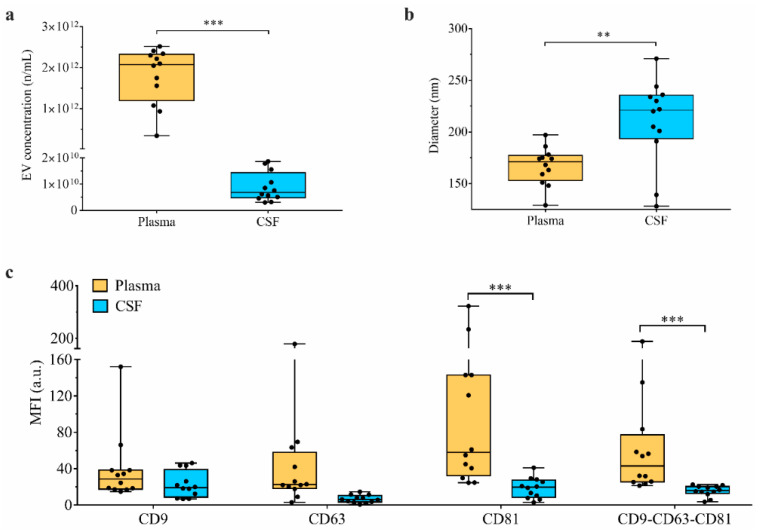
EV quantitative analysis: plasma vs. CSF. Quantitative analysis of extracellular vesicles (EVs) by nanoparticle tracking analysis (NTA) and MACSPlex assay flow cytometry; plasma samples were compared to paired cerebrospinal fluid (CSF) samples in patients with PD (Parkinson’s disease; *n* = 4), MSA (multisystem atrophy; *n* = 4) and AP-Tau (atypical parkinsonism with tauopathies; *n* = 4). (**a**) EV concentration (n/mL plasma or CSF) at NTA. (**b**) EV diameter (nm) at NTA. (**c**) MFI (expressed as arbitrary unity; a.u.) for CD9, CD63, CD81 and CD9-CD63-CD81 at flow cytometry. Boxplots show median and interquartile range; bars show minimum and maximum values (** *p* < 0.05; *** *p* < 0.001). Data and statistics are reported in Table S2.

We compared plasma- and CSF-derived EVs of the same subjects in pathological groups (PD, MSA, and AP-Tau; *n* = 12). Plasma samples showed higher numbers of nanoparticles/mL compared to CSF, even after stratification for EV diameter (*p* < 0.001 for all comparisons; Figure 4a and Table S3). Moreover, CSF-derived nanoparticles were significantly larger (*p* = 0.005; Figure 4b). We quantified median fluorescence intensity (MFI) of tetraspanins CD9, CD63 and CD81 (specific markers of EVs) by flow cytometry as an alternative measure of EV concentration. Plasmatic samples had higher level of CD81-MFI and of average MFI for CD9, CD63 and CD81 (*p* < 0.001), compared to CSF (Figure 4c and Table S3). Consistently, nanoparticle concentration by NTA directly correlated to mean MFI for CD9-CD63-CD81 (R = 0.552; *p* = 0.005). Among the 37 EV surface markers analyzed in paired plasma vs. CSF samples, 5 markers were differentially expressed in the PD group, 11 in MSA, and 8 in AP-Tau. All these markers were more highly expressed in plasma than CSF except for CD9 in the MSA group (Figures S1 and S2 and Table S4).

### 3.5. The Integrated Random Forest Model Demonstrates Higher Diagnostic Accuracy

**Figure 5:**
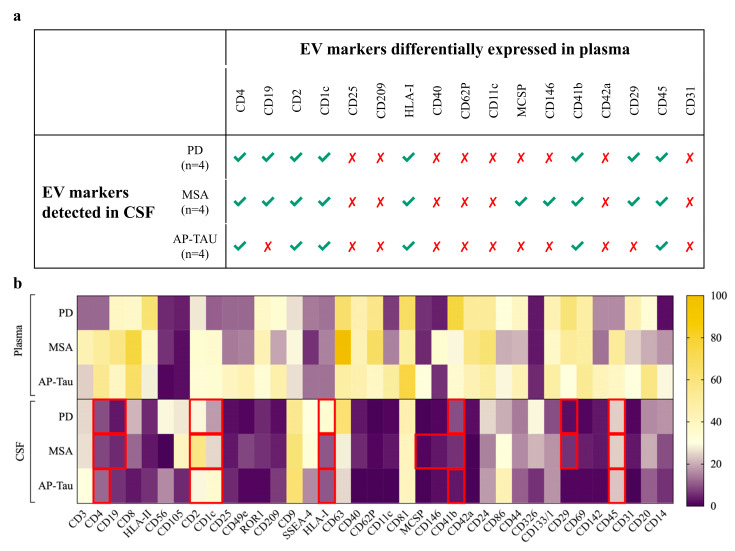
EV surface marker selection. Selection of extracellular vesicle (EV) surface markers for the 2-level random forest diagnostic model: (**a**) EV surface markers differentially expressed in plasma and detected in cerebrospinal fluid (CSF) of Parkinson’s disease (PD), multisystem atrophy (MSA), or atypical parkinsonism with tauopathies (AP-Tau). (**b**) Heatmap representation of the 37 EV surface markers in plasma and CSF of matched subjects with PD, MSA and AP-Tau. The selected markers in each diagnostic category were highlighted with red boxes.

In our previous work, 17 plasmatic EV surface markers were differentially expressed in 29 idiopathic PD, 9 probable MSA, 10 probable AP-Tau patients compared to 36 HC (plasma discovery cohort). A random forest (RF) model (we will refer to this model as “basic” throughout the present manuscript) was built combining levels of expression of these EV markers [14]. Among the 17 EV surface markers differentially expressed in the plasma discovery cohort, we selected for each diagnostic group, those which were expressed also in CSF (nMFI different from 0). Eight were expressed also in CSF-derived EVs in patients with PD (CD4, CD19, CD2, CD1c, HLA-I, CD41b, CD29, and CD45), 10 in patients with MSA (CD4, CD19, CD2, CD1c, HLA-I, MCSP, CD146, CD41b, CD29, and CD45), and 6 in patients with AP-Tau (CD4, CD2, CD1c, HLA-I, CD41b, and CD45) (Figure 5). Assuming that EV surface markers expressed both in plasma and CSF may have higher relevance for the respective neurodegenerative disease, we built an “integrated” version of our basic diagnostic model based on plasma EV expression. In particular, we built one model for each type of disease (PD, MSA, AP-Tau). Integrated versions of RF models were subsequently validated in the original plasma discovery cohort (Figure 2).

**Figure 6:**
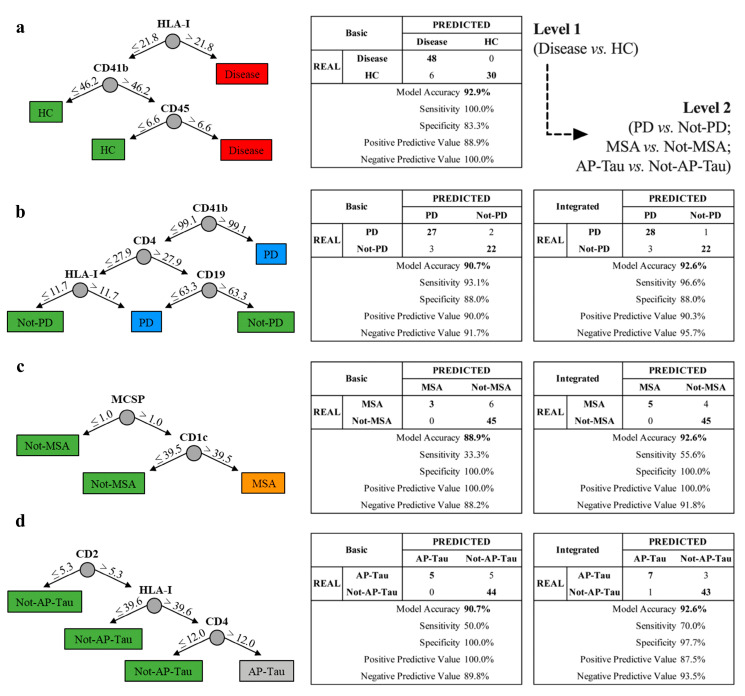
Diagnostic modelling. Development of a 2-level integrated random forest (RF) model to discriminate patients with Parkinson’s disease (PD), multisystem atrophy (MSA), or atypical parkinsonism with tauopathies (AP-Tau) in the plasma discovery cohort (*n* = 84; characteristics of patients are reported in Table S4). (**a**) Level 1 of RF model, discriminating subjects with neurodegenerative diseases from healthy controls (HC). Patients classified as “disease” at level 1 were introduced to level 2. (**b**) Two-level RF model, discriminating PD from non-PD patients. (**c**) Two-level RF model, discriminating MSA from non-MSA patients. (**d**) Two-level RF model, discriminating AP-Tau from non-AP-Tau patients. Confusion matrix (basic vs. integrated version) and a representative classification tree are shown for each RF model. In bold mark are represented the numbers of correctly predicted diagnosis and overall accuracy of the model.

The level 1 RF basic model [14] (built on the 17 EV surface markers differentially expressed in plasma) was applied to the discovery cohort (*n* = 84; Table S1) to differentiate subjects with neurodegenerative diseases from HC (Figure 2). All patients were correctly diagnosed by the model (sensitivity 100.0%), whereas 6 of 36 HC were misclassified (specificity 83.3%). With an overall accuracy of 92.9% (Figure 6a), 54 patients (48 patients and 6 HC) were introduced to level 2 analysis, aiming at the selective recognition of PD, MSA, or AP-Tau. The level 2 RF integrated model discriminated PD from non-PD patients (Figure 6b) with higher sensitivity then the RF basic model (96.6% vs. 93.1%, respectively), and increased accuracy (92.6% vs. 90.7%, respectively). Similarly, the diagnostic performance was higher in the integrated version for discriminating MSA from non-MSA patients (Figure 6c), with an accuracy of 92.6% and a sensitivity of 55.6%, while the RF basic model displayed an accuracy and sensitivity of 88.9% and 33.3%. Finally, the advanced RF model discriminating AP-Tau from non-AP-Tau patients (Figure 6d) correctly predicted 50 of 54 patients (92.6% accuracy) with an increased sensitivity (70.0%) and a specificity comparable to the basic model (97.7%).

Overall, the integrated version of the RF model allowed an increased sensitivity in discriminating specific diseases, with higher negative predictive values for all groups of patients.

## 4. Discussion

The main result of this study is the optimization of a diagnostic model for PD, MSA and AP-Tau based on immunophenotyping of plasma-derived EVs by integrating data from CSF-derived EVs via machine learning algorithms. Our previous basic diagnostic model [14], built on 17 EV surface markers differentially expressed in plasma between groups, correctly predicted patient diagnosis in 88.9% of subjects. The new optimized two-level RF integrated diagnostic model displayed an overall improved diagnostic accuracy of 92.6%, with an increased sensitivity for all three diagnostic categories with respect to the basic RF model. This result is even more relevant, considering the low numbers of subjects analyzed in this pilot study and opens up the possibility to further improve the accuracy of the model by applying this strategy to larger study groups. Indeed, CSF bears the obvious advantages of being more specific for the CNS environment but at the same time the collecting procedure is more invasive for patients and the protocol to enrich for EVs is more challenging. Thus, our strategy to recalibrate the model on EV surface markers that were expressed both in plasma and CSF in each diagnostic group allowed us to improve the diagnostic system that remains based on EV profiling in plasma. This is a novel approach to blood biomarkers by profiling EV surface markers related to inflammatory and immune cells with potential roles in inflammation related to neurodegeneration, while most of the current studies on EVs as biomarkers are focused especially on evaluating target proteins in neuronal-derived exosomes [24,25]. Further, the multiplexed profiling of inflammation markers allows a more personalized approach, as this biomarker-driven phenotyping might be capable of capturing the clinical heterogeneity of PD and may be used to measure the effect of potential disease-modifying drugs on peripheral inflammatory processes as a proxy for central events. Nevertheless, the direct application of EV immunocapturing and multiarray analysis by flow cytometry to biological fluids (without isolation steps by ultracentrifugation or size exclusion chromatography) has clinical relevance, as it can be achieved in hospital-based diagnostic laboratories. Given its low cost, speed, and simplicity, as well as its high sensitivity and specificity, this approach provides a potential biochemical index to support the clinical assessment of PD and AP. Circulating plasma-derived EVs represent an even more promising tool in characterizing, monitoring, and predicting PD, if their profiling can be achieved avoiding time-consuming protocols and sophisticated instrumentation.

PD patients showed a higher number of EVs in CSF compared to HC, as also demonstrated in plasma [14]. In analogy, MSA and AP-Tau showed a tendency towards higher EV numbers and diameters than HC, but this was not statistically significant, probably due to the low sample size. This result is in line with other observations in patients with Alzheimer’s disease where the amount of EVs correlated to myelin damage and neuronal loss [26] and in a mice model of multiple sclerosis in which EVs correlated to brain inflammation, suggesting a pathological role of EVs [27]. Notably, we observed an increase in EVs in neurodegenerative diseases that are typical of elderly populations, while in physiological conditions a decrease in the number of CSF-EVs has been observed by aging [28]. Several human and animal studies have shown that neurodegenerative disorders are characterized by a relevant inflammatory component. PD patients, for example, display neuroinflammatory changes in brain histopathology as well as by elevated immune markers in peripheral blood [29,30], and inflammation has been correlated to the reduction in tyrosine hydroxylase dopaminergic neurons [31] and the expansion of activated microglia in the substantia nigra in animal models of the disease [32]. In an in vitro study, microglial cells activated by proinflammatory stimuli release more microvesicles than exosomes [33]; thus, the higher amount of EVs that we observed in CSF of PD could be related to the inflammatory component.

Patients with PD, MSA and AP-Tau showed a tendency towards larger EVs in the CSF compared to HC, and moreover, in matched CSF and plasma samples of same patients, CSF-derived EVs showed larger EVs compared to the plasmatic one. Thus, it could be conceivable that in disease groups there is a different pathway of vesicle generation towards production of microvesicles (50–1000 nm diameters), generated via outward budding of the plasma membrane, rather than exosomes (40–120 nm) produced in multivesicular bodies via endosomal pathway [34]. Indeed, recent observations suggest that microvesicles may have a role in inflammatory diseases [35] and it has been proposed that they act as a bridge between inflammation and neurodegeneration [36]. An in-depth EV characterization, with different methodologies and with special regard to markers differentially expressed by microvesicles and exosomes, is required to confirm this observation [35].

Regarding the EV immune profiling by MACSPlex human exosome assay applied to CSF-derived EVs, no statistically significant differences were observed in the 37 surface markers between groups. This is certainly due to the main limitation of this explorative study which is the low number of subjects analyzed in each diagnostic category. However, the simultaneous quantification of multiple EV markers increased the power of the assay and displayed different EV profiles of expression in subjects with PD and AP vs. HC, which allowed a good discrimination of patients in accordance with the clinical diagnosis by linear discriminant analysis. Of interest, PD and AP patients expressed higher amounts of HLA-I, CD8, CD2, CD3, CD14 and CD20 in CSF, while no or very low detection was observed in HC, suggesting a CNS activation of the immune system, in particular of the T cell-mediated immunity with particular emphasis on intracellular endogenous synthetized antigens that involves the activation of HLA class I [37,38] and are presented to CD8 T cells [39]. Indeed, a recent work on human substantia nigra demonstrated that CD8 T cell infiltration is an early pathogenic event and parallels the progression of neuronal loss and alpha synuclein aggregation in PD [40]. This is particularly relevant in diseases which are pathologically characterized by intracellular accumulation of misfolded proteins and confirms the crucial role which EVs play in antigen presenting immunity by spreading HLA proteins, possibly increasing the number of dendritic cells or phagocytes presenting them or directly interacting with T cells [41].

CD105 is highly expressed in CSF of PD and MSA. Even if it is used as an endothelial cell marker during angiogenesis [42], it was originally described as a marker for activated macrophages and can identify subtypes of activated microglia [43]. In fact, in the substantia nigra of PD subjects a strong CD105 staining in microglia cells was described in association with degenerating dopaminergic neurons [44]. Activation of microglia is supported also by expression of CD86 in AP-Tau and MSA, whose upregulation had consistently been associated with activated microglia [45], and to a lesser extent in neurons [46] and astrocytes [47], during inflammatory processes.

Finally, levodopa represents the first-line therapy in PD. Several drugs may influence the mechanism of biogenesis and release of EVs [48]; however, to our knowledge, a direct effect of levodopa has never been demonstrated. Indeed, in our previous study, we did not find any significant association when we correlated EV parameters with Levodopa Equivalent Daily Dose (LEDD) in PD and AP subjects [14]. However, larger studies on this topic are certainly recommended.

This is an explorative study and the low number of subjects in the CSF cohort is certainly a limitation; therefore, larger studies and validation in external cohorts of patients are warranted. In addition, it is an antemortem study, lacking the diagnostic confirmation of brain histopathologic analysis. Finally, although it is beyond the primary scope of this paper, a deeper characterization of CSF-derived EVs with different techniques such as transmission electron microscopy (TEM), would be insightful. However, the enrichment of vesicle fraction by immunocapturing displays an inherent limitation for TEM analysis; in fact, a bead–EV complex might impair the ultrastructural morphological characterization of nanosized vesicles. Nevertheless, we can speculate that at least for plasma-derived EVs, by avoiding ultracentrifugation, which is known to lead to EV deformation and rapture into smaller particles [49], the morphological structure of immuno-captured EVs is preserved.

In conclusion, a diagnostic model based on plasmatic EVs, built via machine learning, integrating information provided by the simultaneous profiling of CSF and plasma could potentially impact on clinical practice. Furthermore, the EV surface markers characterization bolsters the concept of a relevant involvement of inflammation in PD and it underscores the importance of EVs as pathways/biomarkers for protein aggregation-related neurodegenerative diseases.

## Figures and Tables

**Table 1:** Clinical characteristics of the cerebrospinal fluid (CSF) optimization cohort.

Variable	HC (*n* = 4)	PD (*n* = 4)	AP		*p*-Value
			MSA (*n* = 4)	AP-Tau (*n* = 4)	
Age (years)	59 ± 20.3	67 ± 12.9	60 ± 4.6	66 ± 12.5	0.794
Sex (ref. male)	1 (25.0)	2 (50.0)	1 (25.0)	2 (50.0)	0.785
Disease duration (years)	-	4.0 [0.8–6.5]	3.0 [1.0–5.8]	3.5 [3.0–8.5]	0.859
H&Y	-	1.5 [1.0–2.8]	3.5 [2.3–4.8]	4.0 [2.0–4.0]	0.129
MDS-UPDRS	-	34.5 [17.0–51.3]	34.8 [26.5–34.8]	67.0 [29.0–67.0]	0.511
Beck Depression	-	7.0 [2.3–14.0]	5.5 [3.5–9.0]	16.0 [11.0–16.0]	0.094
MMSE	-	30.0 [30.0–30.0]	28.0 [24.5–30.0]	24.0 [22.0–24.0]	0.051
MOCA	-	28.0 [26.3–29.8]	23.0 [16.3–29.0]	19.0 [16.0–19.0]	0.114
Olfactory test	-	6.0 [3.0–6.0]	9.0 [4.5–10.5]	7.0 [5.0–7.0]	0.722
RBD	-	7.0 [3.0–8.8]	4.0 [2.5–4.8]	2.0 [1.0–2.0]	0.139
LEDD	-	238 [100.0–750.0]	-	-	-

Clinical characteristics of healthy controls (HC) compared to patients diagnosed with Parkinson’s disease (PD), multisystem atrophy (MSA), or atypical parkinsonism with tauopathies (AP-Tau). Variables are reported as mean ± SD, median (interquartile range) and absolute number (percentage), as appropriate. Abbreviations: H&Y (Hoehn and Yahr scale), MDS-UPDRS (Movement Disorder Society-Unified Parkinson’s Disease Rating Scale), BDI-II (Beck Depression Inventory II), MMSE (Mini-Mental State Examination), MoCA (Montreal Cognitive Assessment), RBD (Rem Behavior Disorder scale), and LEDD (LevoDopa equivalent Dose).

## Data Availability

Raw data that support the findings of this manuscript are available upon reasonable request to the corresponding author.

## Supplementary Materials

The following are available online at https://www.mdpi.com/2227-9059/9/3/230/s1, Table S1: Characteristics of patients in plasma discovery cohort; Table S2: NTA and MACSPlex evaluation of CSF-derived EVs; Table S3. EV quantitative analysis: plasma and CSF; Table S4. EV surface antigens in plasma and CSF in pathological groups; Figure S1. EV surface marker expression in plasma and CSF; Figure S2. Evaluation of EV surface antigens in pathological groups.
