# Supplementary material for: Profiling Inflammatory Extracellular Vesicles in Plasma and Cerebrospinal Fluid: An Optimized Diagnostic Model for Parkinson’s Disease
Source: Biomedicines. 2021 Feb 25;9(3):230. doi: 10.3390/biomedicines9030230 (PMC7996605; doi:10.3390/biomedicines9030230)
Supplement: Supplementary file 1 [file biomedicines-09-00230-s001.pdf]

*Supplementary material*

# Profiling inflammatory extracellular vesicles in plasma and cerebrospinal fluid: an optimized diagnostic model for Parkinson's disease

**Elena Vacchi[1,2,#], Jacopo Burrello[3,#], Alessio Burrello[4], Sara Bolis[3], Silvia Monticone[5], Lucio Barile[2,3], Alain Kaelin-Lang[1,2,6,7] , Giorgia Melli[1,2,6,*]**

1. Laboratory for Biomedical Neurosciences, Neurocenter of Southern Switzerland, Ente Ospedaliero Cantonale, Lugano, Switzerland. elena.vacchi@eoc.ch (E.V.)
2. Faculty of Biomedical Sciences, Universita della Svizzera Italiana; Lugano, Switzerland. lucio.barile@cardiocentro.org (L.B.)
3. Laboratory for Cardiovascular Theranostics, Cardiocentro Ticino Foundation, Lugano, Switzerland. Jacopo.burrello@gmail.com (J.B.), sara.bolis@cardiocentro.org (S.B.)
4. Department of Electrical, Electronic and Information Engineering "Guglielmo Marconi", University of Bologna, Bologna, Italy. alessio.burrello@unibo.it (A.B.)
5. Division of Internal Medicine and Hypertension Unit, Department of Medical Sciences, University of Torino, Torino, Italy. smv.monticone@gmail.com (S.M.)
6. Neurology Department, Neurocenter of Southern Switzerland, Ente Ospedaliero Cantonale, Lugano.
7. Department of Neurology, Inselspital, Berne University Hospital, Berne, Switzerland. alain.kaelin@eoc.ch (A.K-L.)

\# Contributed equally and should be considered as joint first authors

* Correspondence: giorgia.melli@eoc.ch (G.M.); Tel.+41-(0)918116535; Fax:+41-(0)91-8116915

Summary:

Table S1. Characteristics of patients in plasma discovery cohort

Table S2. NTA and MACSPlex evaluation of CSF-derived EVs

Table S3. EV quantitative analysis: plasma and CSF

Figure S1. EV surface markers expression in plasma and CSF

Figure S2. Evaluation of EV surface antigens in pathological groups

Table S4. EV surface antigens in plasma and CSF in pathological groups

**Table S1.** Characteristics of patients in plasma discovery cohort

| **Variable** | **HC** [n=36] | **PD** [n=29] | **AP** | | **Overall *P*-value** | ***Pairwise Comparisons*** | | | | | |
|---|---|---|---|---|---|---|---|---|---|---|---|
| | | | **MSA** [n=9] | **AP-Tau** [n=10] | | HC vs. PD | HC vs. MSA | HC vs. AP-Tau | PD vs. MSA | PD vs. AP-Tau | MSA vs. AP-Tau |
| Age (years) | 62 ± 8.6 | 67 ± 11.6 | 66 ± 8.7 | 72 ± 8.6 | **0.025** | 0.341 | 1.000 | **0.027** | 1.000 | 0.774 | 1.000 |
| Sex (ref. male) | 18 (50.0) | 18 (62.1) | 3 (33.3) | 5 (50.0) | 0.474 | - | - | - | - | - | - |
| Disease duration (years) | - | 4.0 [2.5-8.5] | 5.0 [1.0-7.5] | 3.5 [2.8-5.3] | 0.682 | - | - | - | - | - | - |
| H&Y | - | 2.0 [1.0-3.0] | 4.5 [3.3-5.0] | 4.0 [3.0-5.0] | **<0.001** | - | - | - | **0.001** | **0.001** | 1.000 |
| MDS-UPDRS | - | 24.0 [13.5-37.3] | 42.0 [26.5-42.0] | 47.0 [30.5-99.0] | **0.029** | - | - | - | 0.568 | **0.041** | 1.000 |
| Beck Depression | - | 5.0 [3.0-8.0] | 6.0 [3.0-14.0] | 15.0 [12.5-18.0] | **0.003** | - | - | - | 1.000 | **0.002** | 0.151 |
| MMSE | - | 30.0 [29.0-30.0] | 27.5 [24.3-29.8] | 27.0 [22.5-28.0] | **<0.001** | - | - | - | 0.061 | **0.001** | 0.699 |
| MOCA | - | 27.0 [23.3-29.0] | 23.0 [18.0-26.0] | 19.5 [14.5-22.5] | **0.006** | - | - | - | 0.292 | **0.008** | 0.726 |
| Olfactory test | - | 7.0 [4.0-8.6] | 9.0 [6.0-10.0] | 7.0 [4.5-8.5] | 0.305 | - | - | - | - | - | - |
| RBD | - | 3.0 [2.0-5.0] | 4.0 [1.0-5.5] | 2.5 [0.8-4.3] | 0.678 | - | - | - | - | - | - |
| LEDD | - | 563 [180-750] | - | - | - | - | - | - | - | - | - |

Clinical characteristics of patients from the plasma discovery cohort (n=84): healthy controls (HC) are compared to patients with PD (Parkinson's disease), MSA (multisystem atrophy), or AP-Tau (atypical parkinsonism with tauopathies). Variables are reported as mean ± SD, median [interquartile range], absolute number (percentage), as appropriated. A $P$<0.05 was considered significant and shown in bold.

Abbreviations: H&Y (Hoehn and Yahr scale), MDS-UPDRS (Movement Disorder Society-Unified Parkinson's Disease Rating Scale), BDI-II (Beck Depression Inventory II), MMSE (Mini-Mental State Examination), MoCA (Montreal Cognitive Assessment), RBD (Rem Behavior Disorder scale), LEDD (LevoDopa equivalent Dose).

**Table S2.** NTA and MACSPlex evaluation of CSF-derived EVs

| Variable | HC [n=4] | PD [n=4] | AP: MSA [n=4] | AP: AP-Tau [n=4] | Overall *P*-value | HC vs. PD | HC vs. MSA | HC vs. AP-Tau | PD vs. MSA | PD vs. AP-Tau | MSA vs. AP-Tau |
|---|---|---|---|---|---|---|---|---|---|---|---|
| N° of EVs/mL*E9 (all vesicles) | 0.9 [0.7-1.6] | 7.4 [5.8-10.2] | 9.4 [3.1-17.3] | 6.2 [4.7-15.9] | **0.036** | **0.048** | 0.155 | 0.105 | 1.000 | 1.000 | 1.000 |
| N° of EVs/mL*E9 (30-150nm) | 0.5 [0.4-0.6] | 2.2 [2.0-3.2] | 2.8 [0.6-11.2] | 2.4 [1.2-4.8] | 0.065 | - | - | - | - | - | - |
| N° of EVs/mL*E9 (151-500nm) | 0.4 [0.3-1.1] | 5.4 [3.5-7.0] | 3.6 [2.3-9.2] | 3.6 [2.6-12.4] | **0.033** | **0.045** | 0.188 | 0.128 | 1.000 | 1.000 | 1.000 |
| Diameter (nm) | 155 [135-188] | 211 [194-228] | 228 [160-262] | 221 [147-242] | 0.203 | - | - | - | - | - | - |
| CD9 (MFI; FC) | 4.8 [3.5-7.1] | 18.1 [9.5-39.2] | 24.0 [10.7-39.3] | 16.2 [8.6-37.5] | 0.080 | - | - | - | - | - | - |
| CD63 (MFI; FC) | 3.3 [2.6-5.9] | 9.6 [6.7-13.7] | 3.6 [2.2-9.6] | 4.7 [1.4-7.2] | 0.142 | - | - | - | - | - | - |
| CD81 (MFI; FC) | 14.9 [12.6-19.9] | 22.2 [9.4-28.4] | 16.8 [9.1-26.2] | 15.6 [4.8-36.0] | 0.936 | - | - | - | - | - | - |
| Mean MFI CD9, CD63, CD81 (FC) | 7.7 [6.3-10.9] | 15.7 [14.5-18.7] | 17.8 [7.9-21.5] | 16.3 [5.7-22.1] | 0.281 | - | - | - | - | - | - |
| CD3 (nMFI; %) | 0.0 [0.0-0.0] | 13.6 [3.8-41.2] | 20.8 [15.2-24.7] | 11.7 [0.2-58.2] | 0.052 | - | - | - | - | - | - |
| CD4 (nMFI; %) | 2.7 [1.2-14.9] | 7.4 [0.8-28.8] | 11.0 [1.2-20.1] | 1.7 [0.0-45.1] | 0.909 | - | - | - | - | - | - |
| CD19 (nMFI; %) | 4.6 [1.0-9.5] | 0.8 [0.0-6.0] | 1.0 [0.0-12.3] | 0.0 [0.0-15.7] | 0.783 | - | - | - | - | - | - |
| CD8 (nMFI; %) | 0.0 [0.0-6.5] | 13.5 [2.5-30.9] | 8.2 [0.8-17.0] | 2.1 [0.0-4.5] | 0.162 | - | - | - | - | - | - |
| HLA-II (nMFI; %) | 3.4 [0.0-9.2] | 4.2 [2.1-14.2] | 3.5 [0.0-11.7] | 9.6 [2.1-15.1] | 0.667 | - | - | - | - | - | - |
| CD56 (nMFI; %) | 0.0 [0.0-0.0] | 0.0 [0.0-178.0] | 0.0 [0.0-0.0] | 0.0 [0.0-95.2] | 0.542 | - | - | - | - | - | - |
| CD105 (nMFI; %) | 4.1 | 306.4 | 297.9 | 19.6 | 0.228 | - | - | - | - | - | - |

| | | | | | | | | | | | |
|---|---|---|---|---|---|---|---|---|---|---|---|
| | [0.6-6.2] | [44.7-771.2] | [52.6-1,330.6] | [1.1-188.8] | | | | | | | |
| CD2 (nMFI; %) | 0.0 [0.0-0.0] | 2.9 [0.1-22.7] | 15.3 [4.1-30.6] | 6.3 [0.9-18.1] | 0.059 | - | - | - | - | - | - |
| CD1c (nMFI; %) | 0.0 [0.0-5.9] | 11.4 [0.3-28.0] | 20.3 [9.3-25.0] | 9.9 [3.2-63.7] | 0.165 | - | - | - | - | - | - |
| CD25 (nMFI; %) | 1.2 [0.0-5.7] | 0.0 [0.0-3.9] | 0.0 [0.0-7.9] | 0.0 [0.0-12.8] | 0.935 | - | - | - | - | - | - |
| CD49e (nMFI; %) | 0.0 [0.0-0.0] | 0.0 [0.0-1.7] | 1.2 [0.0-10.1] | 0.0 [0.0-0.5] | 0.366 | - | - | - | - | - | - |
| ROR1 (nMFI; %) | 4.7 [0.8-25.8] | 2.1 [0.1-18.3] | 10.6 [2.3-13.7] | 1.1 [0.0-4.8] | 0.614 | - | - | - | - | - | - |
| CD209 (nMFI; %) | 1.9 [0.0-27.9] | 0.0 [0.0-4.2] | 0.0 [0.0-10.7] | 0.0 [0.0-16.9] | 0.846 | - | - | - | - | - | - |
| CD9 (nMFI; %) | 61.3 [51.3-69.8] | 109.5 [57.6-238.0] | 135.4 [120.9-197.2] | 178.5 [87.1-198.8] | 0.147 | - | - | - | - | - | - |
| SSEA-4 (nMFI; %) | 4.5 [2.5-14.4] | 4.1 [0.8-356.5] | 155.5 [32.2-203.5] | 25.9 [8.4-163.2] | 0.162 | - | - | - | - | - | - |
| HLA-I (nMFI; %) | 0.0 [0.0-40.5] | 22.9 [2.8-217.6] | 11.3 [0.0-57.3] | 13.8 [0.0-54.9] | 0.702 | - | - | - | - | - | - |
| CD63 (nMFI; %) | 45.3 [39.7-53.5] | 60.5 [45.8-73.3] | 28.0 [19.8-48.1] | 27.5 [16.7-45.2] | 0.065 | - | - | - | - | - | - |
| CD40 (nMFI; %) | 0.0 [0.0-2.7] | 0.0 [0.0-7.1] | 0.0 [0.0-12.2] | 0.0 [0.0-0.0] | 0.756 | - | - | - | - | - | - |
| CD62P (nMFI; %) | 0.0 [0.0-0.0] | 0.0 [0.0-0.0] | 0.0 [0.0-7.2] | 0.0 [0.0-0.0] | 0.392 | - | - | - | - | - | - |
| CD11c (nMFI; %) | 3.1 [1.1-6.4] | 0.0 [0.0-0.0] | 0.0 [0.0-9.5] | 0.0 [0.0-0.7] | 0.149 | - | - | - | - | - | - |
| CD81 (nMFI; %) | 194.5 [180.9-203.7] | 131.3 [60.6-183.4] | 131.8 [81.0-137.9] | 89.4 [82.7-174.1] | 0.185 | - | - | - | - | - | - |
| MCSP (nMFI; %) | 0.0 [0.0-5.9] | 0.0 [0.0-0.0] | 5.1 [0.0-11.8] | 0.0 [0.0-4.9] | 0.366 | - | - | - | - | - | - |
| CD146 (nMFI; %) | 2.1 [0.0-4.5] | 0.0 [0.0-5.8] | 0.8 [0.0-12.0] | 0.0 [0.0-7.6] | 0.905 | - | - | - | - | - | - |
| CD41b (nMFI; %) | 0.0 [0.0-0.4] | 4.2 [0.0-25.4] | 2.1 [0.0-18.0] | 2.8 [0.6-5.7] | 0.516 | - | - | - | - | - | - |
| CD42a (nMFI; %) | 0.8 | 0.0 | 0.0 | 0.0 | 0.532 | - | - | - | - | - | - |

| | | | | | | | | | | | |
|---|---|---|---|---|---|---|---|---|---|---|---|
| | [0.0-8.6] | [0.0-9.6] | [0.0-0.0] | [0.0-1.4] | | | | | | | |
| CD24 (nMFI; %) | 9.9 [4.3-45.4] | 10.0 [4.7-41.6] | 11.9 [2.7-18.4] | 7.3 [0.0-33.5] | 0.902 | - | - | - | - | - | - |
| CD86 (nMFI; %) | 0.0 [0.0-2.9] | 4.4 [0.0-47.6] | 21.2 [18.3-41.4] | 24.2 [3.3-85.4] | 0.113 | - | - | - | - | - | - |
| CD44 (nMFI; %) | 0.6 [0.0-8.1] | 9.4 [0.0-30.9] | 16.4 [7.4-22.0] | 2.9 [0.0-29.3] | 0.410 | - | - | - | - | - | - |
| CD326 (nMFI; %) | 0.0 [0.0-7.1] | 0.0 [0.0-840.7] | 121.3 [27.3-468.5] | 48.4 [0.2-135.5] | 0.130 | - | - | - | - | - | - |
| CD133/1 (nMFI; %) | 2.4 [0.7-20.1] | 8.1 [1.8-12.8] | 8.5 [0.3-34.3] | 10.4 [2.0-29.4] | 0.962 | - | - | - | - | - | - |
| CD29 (nMFI; %) | 3.7 [0.0-7.4] | 1.3 [0.0-5.2] | 2.7 [0.0-16.0] | 0.0 [0.0-1.5] | 0.652 | - | - | - | - | - | - |
| CD69 (nMFI; %) | 0.0 [0.0-0.0] | 0.0 [0.0-1.4] | 0.0 [0.0-10.5] | 0.0 [0.0-1.5] | 0.756 | - | - | - | - | - | - |
| CD142 (nMFI; %) | 0.3 [0.0-10.4] | 0.5 [0.0-17.4] | 3.2 [0.0-15.5] | 0.1 [0.0-7.5] | 0.967 | - | - | - | - | - | - |
| CD45 (nMFI; %) | 0.0 [0.0-0.6] | 4.6 [0.2-61.5] | 22.5 [17.6-26.2] | 14.5 [0.4-51.1] | 0.104 | - | - | - | - | - | - |
| CD31 (nMFI; %) | 0.0 [0.0-8.7] | 0.0 [0.0-5.0] | 0.0 [0.0-9.5] | 0.0 [0.0-0.4] | 0.992 | - | - | - | - | - | - |
| CD20 (nMFI; %) | 1.3 [0.0-9.2] | 10.4 [2.5-28.6] | 17.1 [3.4-30.7] | 8.1 [0.0-20.7] | 0.555 | - | - | - | - | - | - |
| CD14 (nMFI; %) | 0.5 [0.0-4.8] | 17.6 [3.8-111.2] | 24.3 [5.4-41.0] | 0.2 [0.0-47.3] | 0.426 | - | - | - | - | - | - |

The table reports EV parameters derived by nanoparticle tracking analysis (NTA) and MACSPlex flow cytometry assay in cerebrospinal fluid (CSF) of healthy controls (HC) and patients with Parkinson's disease (PD), multisystem atrophy (MSA), or atypical parkinsonism with tauopathies (AP-Tau). We considered EV concentration and diameter at NTA, median fluorescence intensity (MFI) and mean MFI for CD9, CD63, and CD81 at flow cytometry, and normalized MFI (nMFI; %) for 37 EV surface antigens at flow cytometry. Data are expressed as median and interquartile range. A *P*<0.05 was considered significant and shown in bold.

**Table S3.** EV quantitative analysis in plasma and CSF

| **Variable** | **Plasma** [n=12] | **CSF** [n=12] | ***P*-value** |
|---|---|---|---|
| N° of EVs/mL (all vesicles*E11) | 20.8 [12.0-23.3] | 0.07 [0.05-0.14] | **<0.001** |
| N° of EVs/mL (30-150nm *E11) | 9.3 [6.3-12.3] | 0.02 [0.01-0.04] | **<0.001** |
| N° of EVs/mL (151-500nm *E11) | 8.6 [5.9-11.8] | 0.04 [0.03-0.07] | **<0.001** |
| Diameter (nm) | 171 [153-177] | 221 [194-234] | **0.005** |
| CD9 (MFI; FC) | 28.8 [17.5-38.4] | 19.1 [8.6-39.1] | 0.374 |
| CD63 (MFI; FC) | 22.6 [18.4-58.0] | 6.0 [3.2-10.4] | 0.071 |
| CD81 (MFI; FC) | 58.0 [32.4-143.0] | 19.7 [8.4-27.4] | **<0.001** |
| Mean MFI CD9, CD63, CD81 (MFI; FC) | 43.0 [25.4-77.2] | 16.0 [12.7-20.4] | **<0.001** |

Extracellular vesicle (EV) concentration and diameter at nanoparticle tracking analysis (NTA), median fluorescence intensity (MFI) and mean MFI for CD9, CD63, and CD81 after flow cytometry are reported for plasma and cerebrospinal fluid (CSF). Data are expressed as median and interquartile range. $P<0.05$ was considered significant and shown in bold.

**Figure S1.** EV surface markers expression in plasma and CSF

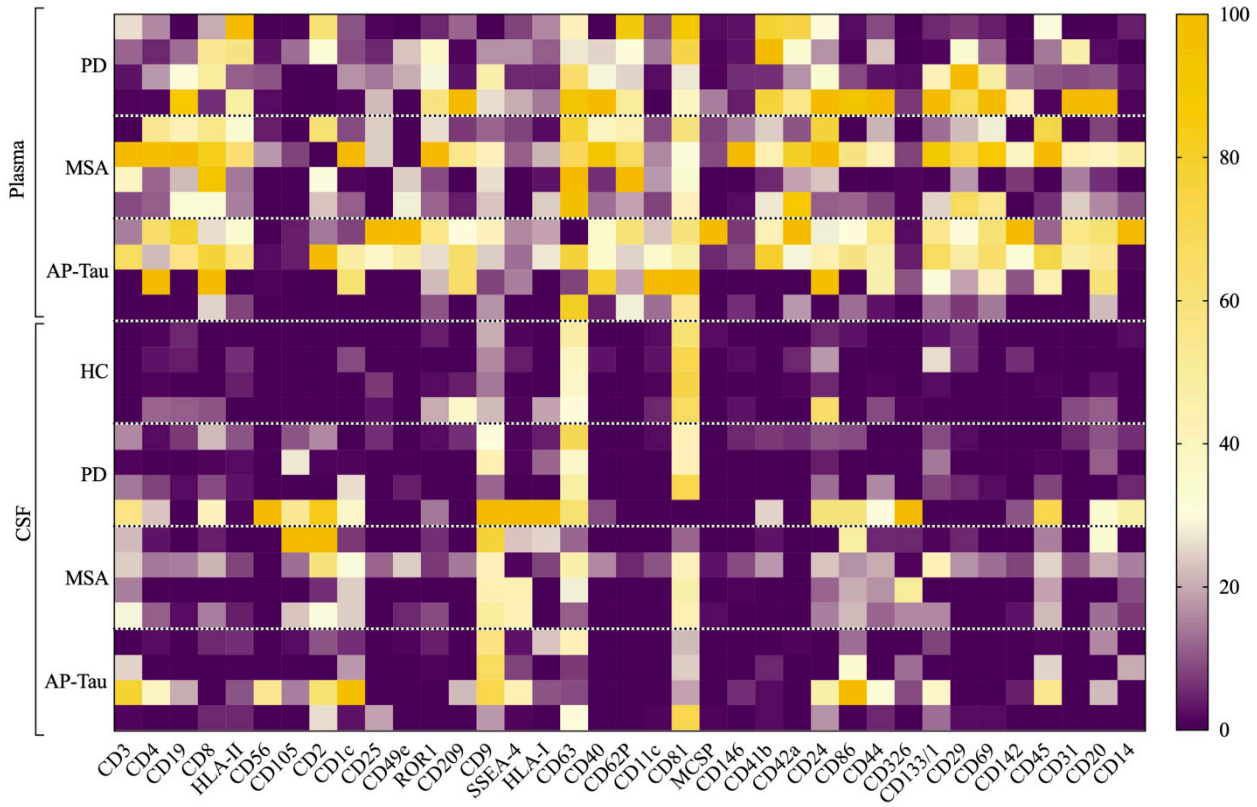


Stratification of patients for diagnosis and expression of extracellular vesicle (EV) surface antigens in plasma and cerebrospinal fluid (CSF). The heat map shows normalized median fluorescence intensity (purple = low nMFI; yellow = high nMFI) for the 28 analyzed samples: 12 plasma samples from patients with PD (Parkinson's disease), MSA (multisystem atrophy), AP-Tau (atypical parkinsonism with tauopathies; n=4 per group) were compared to the respective CSF sample, plus 4 CSF samples from HC.

**Figure S2.** Evaluation of EV surface antigens in pathological groups

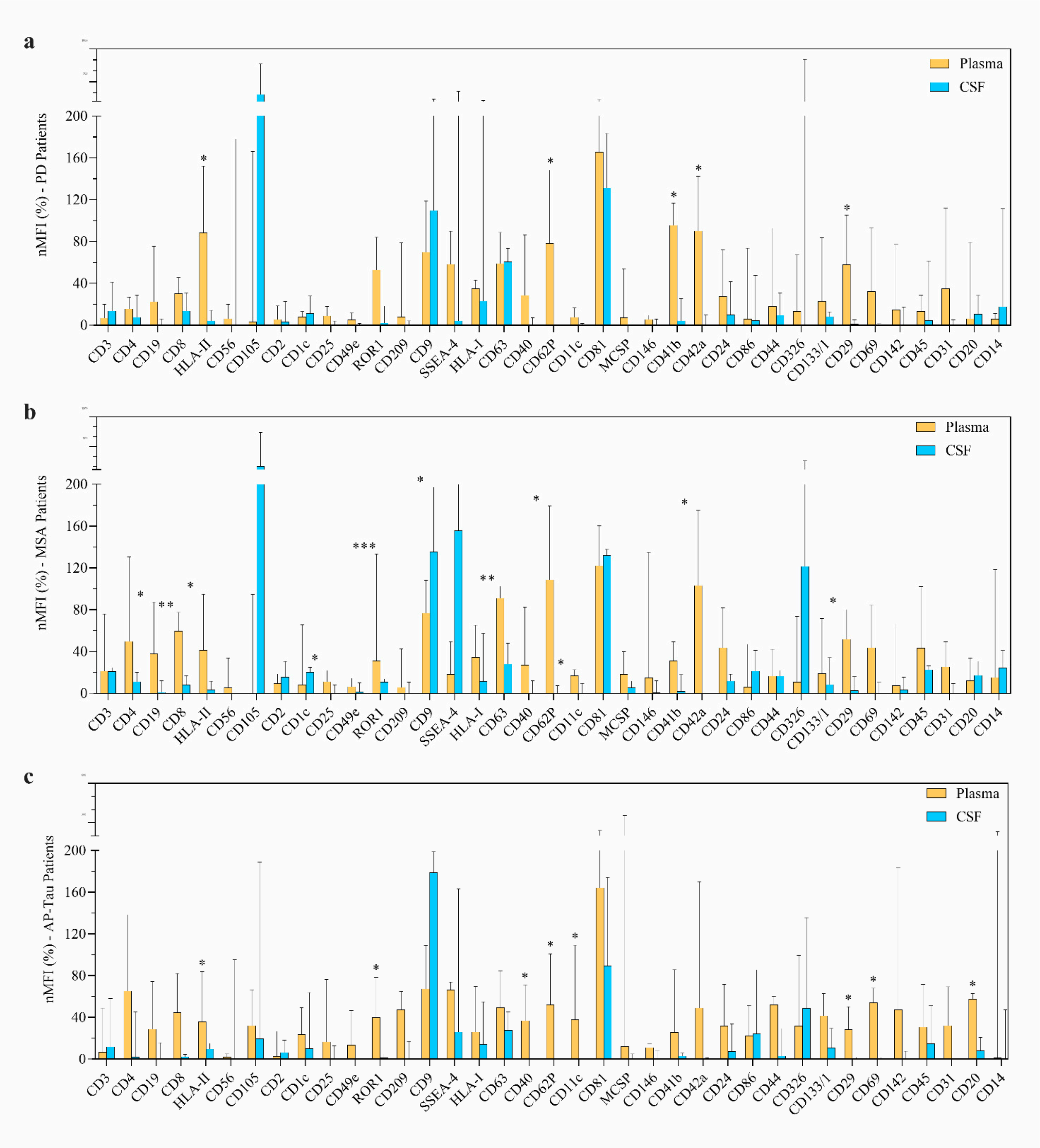


The expression of 37 extracellular vesicle (EV) surface antigens was assessed by flow cytometry and expressed as normalized median fluorescence intensity (nMFI; %); 12 plasma samples (yellow) from patients with PD (Parkinson's disease; **a**), MSA (multisystem atrophy; **b**) and AP-Tau (atypical parkinsonism with tauopathies; **c**; (n=4 per group) were compared to the respective cerebrospinal fluid (CSF; light blue) samples. Bars show median and inter-quartile range (*$P$<0.05; **$P$<0.01; **$P$<0.001). Data and statistics are reported in Table S4.

**Table S4.** EV surface antigens in plasma and CSF in pathological groups

| Variable | **PD** [n=4] | | | **MSA** [n=4] | | | **AP-Tau** [n=4] | | |
|---|---|---|---|---|---|---|---|---|---|
| | **CSF** | **Plasma** | ***P*-value** | **CSF** | **Plasma** | ***P*-value** | **CSF** | **Plasma** | ***P*-value** |
| N° of EVs/mL *E11 (all vesicles) | 0.07 [0.06-0.10] | 19.0 [16.1-20.9] | **0.020** | 0.09 [0.03-0.17.] | 22.6 [12.6-23.8] | **0.021** | 0.06 [0.05-0.16] | 17.1 [5.3-24.8] | **0.025** |
| N° of EVs/mL*E11 (30-150nm) | 0.02 [0.02-0.03] | 9.0 [7.7-11.7] | **0.027** | 0.03 [0.01-0.11] | 10.4 [6.8-12.2] | **0.029** | 0.02 [0.01-0.05] | 8.6 [2.1-16.0] | **0.031** |
| N° of EVs/mL*E11 (151-500nm) | 0.05 [0.04-0.07] | 8.6 [8.4-10.7] | **0.028** | 0.04 [0.02-0.09] | 11.8 [5.5-12.5] | **0.031** | 0.04 [0.03-0.12 | 5.9 [3.2-11.4] | **0.029** |
| Diameter (nm) | 211 [194-228] | 175 [155-183] | **0.033** | 228 [160-262] | 161 [153-171] | 0.343 | 221 [147-242] | 173 [139-192] | 0.343 |
| CD9 (MFI; FC) | 18.1 [9.5-39.2] | 33.7 [22.3-122.7] | 0.200 | 24.0 [10.7-39.3] | 27.8 [17.0-59.2] | 0.886 | 16.2 [8.6-37.5] | 21.5 [15.8-35.0] | 0.686 |
| CD63 (MFI; FC) | 9.6 [6.7-13.7] | 41.8 [18.4-149.7] | **0.028** | 3.6 [2.2-9.6] | 32.5 [22.4-62.5] | **0.029** | 4.7 [1.4-7.2] | 15.5 [4.5-25.0] | 0.200 |
| CD81 (MFI; FC) | 22.2 [9.4-28.4] | 177.4 [48.7-300.4] | 0.114 | 16.8 [9.1-26.2] | 45.4 [26.0-122.5] | 0.057 | 15.6 [4.8-36.0] | 49.9 [41.7-121.0] | 0.057 |
| Mean MFI CD9, CD63, CD81 (FC) | 15.7 [14.5-18.7] | 96.7 [33.5-174.9] | **0.021** | 17.8 [7.9-21.5] | 39.8 [21.9-76.7] | 0.061 | 16.3 [5.7-22.1] | 32.0 [27.3-48.5] | **0.029** |
| CD3 | 13.6 [3.8-41.2] | 6.8 [1.7-20.0] | 0.426 | 20.8 [15.2-24.7] | 21.1 [2.0-76.0] | 0.555 | 11.7 [0.2-58.2] | 6.7 [0.0-48.9] | 0.686 |
| CD4 | 7.4 [0.8-28.8] | 15.7 [2.4-26.9] | 0.801 | 11.0 [1.2-20.1] | 49.7 [17.4-130.6] | 0.129 | 1.7 [0.0-45.1] | 65.3 [8.4-138.4] | 0.343 |
| CD19 | 0.8 [0.0-6.0] | 22.3 [3.4-75.5] | 0.166 | 1.0 [0.0-12.3] | 37.9 [24.8-87.5] | **0.047** | 0.0 [0.0-15.7] | 28.7 [0.0-74.3] | 0.486 |
| CD8 | 13.5 [2.5-30.9] | 30.2 [7.9-45.6] | 0.367 | 8.2 [0.8-17.0] | 59.4 [32.9-77.5] | **0.008** | 2.1 [0.0-4.5] | 44.7 [21.8-82.0] | **0.029** |
| HLA-II | 4.2 [2.1-14.2] | 88.2 [33.5-152.1] | **0.037** | 3.5 [0.0-11.7] | 41.3 [24.2-94.9] | **0.048** | 9.6 [2.1-15.1] | 35.8 [3.2-83.9] | 0.343 |
| CD56 | 0.0 [0.0-178.0] | 6.0 [1.3-20.2] | 0.432 | 0.0 [0.0-0.0] | 5.2 [0.0-33.8] | 0.234 | 0.0 [0.0-95.2] | 1.8 [0.2-5.0] | 0.486 |
| CD105 | 306.4 [44.7-771.2] | 3.0 [0.0-166.1] | 0.162 | 297.9 [52.6-1,330.6] | 0.0 [0.0-94.5] | 0.205 | 19.6 [1.1-188.8] | 31.9 [0.0-66.4] | 1.000 |

| | | | | | | | | | |
|---|---|---|---|---|---|---|---|---|---|
| CD2 | 2.9 [0.1-22.7] | 5.5 [0.0-18.4] | 0.946 | 15.3 [4.1-30.6] | 9.3 [2.0-18.6] | 0.444 | 6.3 [0.9-18.1] | 2.3 [0.0-26.5] | 1.000 |
| CD1c | 11.4 [0.3-28.0] | 7.8 [2.8-13.0] | 0.530 | 20.3 [9.3-25.0] | 8.2 [2.8-65.4] | 0.731 | 9.9 [3.2-63.7] | 23.3 [1.6-49.1] | 1.000 |
| CD25 | 0.0 [0.0-3.9] | 8.9 [2.1-18.0] | 0.104 | 0.0 [0.0-7.9] | 10.9 [0.0-22.0] | 0.268 | 0.0 [0.0-12.8] | 16.5 [0.0-76.4] | 0.486 |
| CD49e | 0.0 [0.0-1.7] | 5.2 [0.0-11.7] | 0.177 | 1.2 [0.0-10.1] | 6.4 [0.0-14.4] | 0.555 | 0.0 [0.0-0.5] | 13.1 [0.0-46.5] | 0.486 |
| ROR1 | 2.1 [0.1-18.3] | 52.9 [17.4-84.2] | 0.051 | 10.6 [2.3-13.7] | 31.1 [15.6-133.2] | 0.197 | 1.1 [0.0-4.8] | 39.9 [21.0-78.5] | **0.029** |
| CD209 | 0.0 [0.0-4.2] | 7.7 [1.8-78.7] | 0.287 | 0.0 [0.0-10.7] | 5.6 [1.0-42.4] | 0.370 | 0.0 [0.0-16.9] | 47.3 [7.6-64.8] | 0.114 |
| CD9 | 109.5 [57.6-238.0] | 69.9 [25.1-118.7] | 0.292 | 135.4 [120.9-197.2] | 76.8 [52.6-108.2] | **0.034** | 178.5 [87.1-198.8] | 97.3 [40.1-129.1] | 0.114 |
| SSEA-4 | 4.1 [0.8-356.5] | 58.2 [26.2-89.9] | 0.619 | 155.5 [32.2-203.5] | 18.2 [0.2-49.4] | 0.069 | 25.9 [8.4-163.2] | 66.0 [15.6-73.9] | 0.886 |
| HLA-I | 22.9 [2.8-217.6] | 34.8 [17.7-43.2] | 0.487 | 11.3 [0.0-57.3] | 34.3 [6.8-65.1] | 0.603 | 13.8 [0.0-54.9] | 25.8 [0.0-69.7] | 0.868 |
| CD63 | 60.5 [45.8-73.3] | 58.8 [37.6-88.8] | 0.907 | 28.0 [19.8-48.1] | 91.0 [76.6-102.4] | **0.001** | 27.5 [16.7-45.2] | 49.1 [11.3-84.4] | 0.886 |
| CD40 | 0.0 [0.0-7.1] | 28.5 [6.6-86.3] | 0.143 | 0.0 [0.0-12.2] | 26.9 [7.9-82.5] | 0.146 | 0.0 [0.0-0.0] | 36.9 [12.6-71.2] | **0.029** |
| CD62P | 0.0 [0.0-0.0] | 78.0 [51.9-148.2] | **0.012** | 0.0 [0.0-7.2] | 108.5 [49.8-179.2] | **0.017** | 0.0 [0.0-0.0] | 51.9 [39.9-100.8] | **0.031** |
| CD11c | 0.0 [0.0-0.0] | 7.4 [0.8-16.4] | 0.091 | 0.0 [0.0-9.5] | 16.8 [10.5-22.7] | **0.025** | 0.0 [0.0-0.7] | 37.5 [20.6-109.1] | **0.030** |
| CD81 | 131.3 [60.6-183.4] | 165.5 [99.6-231.0] | 0.410 | 131.8 [81.0-137.9] | 121.9 [110.2-160.3] | 0.555 | 89.4 [82.7-174.1] | 164.0 [144.4-241.6] | 0.200 |
| MCSP | 0.0 [0.0-0.0] | 7.3 [5.2-54.0] | 0.208 | 5.1 [0.0-11.8] | 18.1 [0.0-39.9] | 0.286 | 0.0 [0.0-4.9] | 12.1 [0.6-356.4] | 0.200 |
| CD146 | 0.0 [0.0-5.8] | 5.4 [4.5-9.5] | 0.112 | 0.8 [0.0-12.0] | 14.7 [0.8-134.8] | 0.305 | 0.0 [0.0-7.6] | 10.8 [2.6-15.0] | 0.114 |
| CD41b | 4.2 [0.0-25.4] | 95.4 [28.9-116.7] | **0.037** | 2.1 [0.0-18.0] | 31.0 [12.1-49.4] | 0.076 | 2.8 [0.6-5.7] | 25.6 [0.0-85.7] | 0.886 |
| CD42a | 0.0 | 90.1 | **0.014** | 0.0 | 103.0 | **0.045** | 0.0 | 49.1 | 0.114 |

| | | | | | | | | | |
|---|---|---|---|---|---|---|---|---|---|
| | [0.0-9.6] | [43.0-142.6] | | [0.0-0.0] | [25.0-175.1] | | [0.0-1.4] | [9.4-170.0] | |
| CD24 | 10.0 [4.7-41.6] | 27.3 [17.6-72.0] | 0.342 | 11.9 [2.7-18.4] | 43.4 [12.6-81.9] | 0.114 | 7.3 [0.0-33.5] | 31.6 [6.2-71.6] | 0.486 |
| CD86 | 4.4 [0.0-47.6] | 5.9 [0.9-73.6] | 0.739 | 21.2 [18.3-41.4] | 6.0 [0.0-47.0] | 0.573 | 24.2 [3.3-85.4] | 22.0 [3.3-51.1] | 0.686 |
| CD44 | 9.4 [0.0-30.9] | 18.1 [4.7-92.7] | 0.392 | 16.4 [7.4-22.0] | 16.6 [2.3-42.2] | 0.667 | 2.9 [0.0-29.3] | 51.9 [15.4-60.2] | 0.114 |
| CD326 | 0.0 [0.0-840.7] | 13.0 [2.1-67.2] | 0.403 | 121.3 [27.3-468.5] | 10.8 [1.9-73.7] | 0.211 | 48.4 [0.2-135.5] | 31.8 [8.2-99.4] | 0.886 |
| CD133/1 | 8.1 [1.8-12.8] | 22.5 [1.5-83.7] | 0.260 | 8.5 [0.3-34.3] | 18.8 [3.1-71.5] | 0.464 | 10.4 [2.0-29.4] | 41.5 [16.9-62.8] | 0.200 |
| CD29 | 1.3 [0.0-5.2] | 57.8 [15.5-105.3] | **0.049** | 2.7 [0.0-16.0] | 51.6 [21.6-80.1] | **0.038** | 0.0 [0.0-1.5] | 28.2 [11.1-50.1] | **0.029** |
| CD69 | 0.0 [0.0-1.4] | 32.2 [6.5-92.9] | 0.112 | 0.0 [0.0-10.5] | 43.5 [7.5-84.3] | 0.085 | 0.0 [0.0-1.5] | 53.8 [22.1-68.0] | **0.026** |
| CD142 | 0.5 [0.0-17.4] | 14.5 [0.0-77.3] | 0.321 | 3.2 [0.0-15.5] | 7.2 [0.0-66.6] | 0.406 | 0.1 [0.0-7.5] | 47.1 [5.7-183.6] | 0.200 |
| CD45 | 4.6 [0.2-61.5] | 13.1 [3.8-28.9] | 0.747 | 22.5 [17.6-26.2] | 43.2 [1.8-102.1] | 0.359 | 14.5 [0.4-51.1] | 30.6 [3.4-71.7] | 0.686 |
| CD31 | 0.0 [0.0-5.0] | 34.7 [2.9-112.0] | 0.154 | 0.0 [0.0-9.5] | 25.2 [4.8-49.2] | 0.100 | 0.0 [0.0-0.4] | 32.1 [0.0-69.4] | 0.486 |
| CD20 | 10.4 [2.5-28.6] | 6.1 [0.5-79.1] | 0.586 | 17.1 [3.4-30.7] | 11.9 [6.8-33.7] | 0.975 | 8.1 [0.0-20.7] | 57.4 [30.4-62.9] | **0.029** |
| CD14 | 17.6 [3.8-111.2] | 5.9 [0.5-11.1] | 0.288 | 24.3 [5.4-41.0] | 15.0 [0.0-118.3] | 0.588 | 0.2 [0.0-47.3] | 1.0 [0.0-231.6] | 0.885 |

The table reports plasma and cerebrospinal fluid (CSF)-derived extracellular vesicle (EV) parameters derived by nanoparticle tracking analysis (NTA) and flow cytometry for patients with PD (Parkinson's disease), MSA (multisystem atrophy), or AP-Tau (atypical parkinsonism with tauopathies). We considered EV concentration and diameter at NTA, median fluorescence intensity (MFI) and mean MFI for CD9, CD63, and CD81 at flow cytometry, and normalized MFI (nMFI; %) for 37 EV surface antigens at flow cytometry. Data are expressed as median and interquartile range. A $P<0.05$ was considered significant and shown in bold.
